# Supplementary material for: Polymer-coated urea effects on yield and gaseous loss of nitrogen for planting Chinese cabbage
Source: PLoS One. 2025 Nov 26;20(11):e0337171. doi: 10.1371/journal.pone.0337171 (PMC12654927; doi:10.1371/journal.pone.0337171)
Supplement: Attachment — Submitted filename: Response to Reviewers.docx [file pone.0337171.s003.docx]

**Response to Reviewers**

Dear Editor and Reviewers,

Thanks for your comments on our manuscript entitled “Polymer-coated urea effects on yield and gaseous loss of nitrogen for planting Chinese cabbage” (ID: PONE-D-25-22820). We appreciate the opportunity to revise our manuscript and respond to the comments provided by the editors and reviewers. We have carefully considered all the feedback and have made significant revisions to the manuscript accordingly. Below are responses to the comments from the editor and reviewers one by one.

Response to Editorial Comments:

1.Style and formatting requirements:
We have carefully revised the manuscript to ensure it fully complies with PLOS ONE’s style requirements, including file naming conventions. The text and formatting have been adjusted according to the provided templates.

2.Overlapping text:
We thank the editor for highlighting the overlapping text issues. We have thoroughly reviewed the mentioned publications and have ensured that all sources are appropriately cited. The overlapping text outside the Methods section has been either quoted or rephrased to ensure originality and proper attribution. All relevant references are now included in the manuscript.

3.Financial disclosure:
As requested, we have amended the Financial Disclosure statement to clarify the role of the funders. The revised statement is as follows:
“The funders had no role in study design, data collection and analysis, decision to publish, or preparation of the manuscript.”
This statement has also been included in the cover letter as instructed.

4.ORCID iD:
We confirm that the corresponding author has a validated ORCID iD, which is properly linked and validated in the Editorial Manager system.

5.Supporting Information captions:
We have included captions for all Supporting Information files at the end of the manuscript and have updated all in-text citations to match accordingly.

6.Citation of recommended publications:
We have reviewed the publications suggested by the reviewers and have cited those that are relevant to our work. However, some recommended publications were not directly relevant to the focus of our study and therefore were not included.

Response to Reviewers' Comments:

Comment 1:

The PCU is described only as "polyurethane-coated, 40-day release". Add specific information about PCU characterization such as coating rate (% w/w), membrane thickness, and ideally a cumulative release curve at 25°C (or cite the manufacturer’s technical sheet). Without these data, it is hard to reproduce the work or compare with other PCU types and effects.

Response:

Thank you for this important suggestion. We have supplemented the description of the PCU with its color, particle size, and initial nutrient release rate. In reviewing the manufacturer's technical documentation, we note that the actual release period is 45 days. We apologize for the inaccuracy in our original description, which was due to an oversight during writing. Unfortunately, the coating rate and membrane thickness are proprietary data not provided by the manufacturer and could not be included. In addition, based on the manufacturer's technical chart, we have plotted the cumulative release curve at 25 ° C, which we will provide as supporting information.

Comment 2:

Include regression equations (exponential or linear-plateau) between N rate and cumulative N_2_O, NH_3_, yield, NUE. This will strengthen the dose-response discussion.

Response:

We appreciate this constructive suggestion. Following your advice, we performed regression analyses in SPSS with N rate as the independent variable and cumulative N_2_O, NH_3_, yield, and NUE as dependent variables. Based on scatter plots and model fit, exponential models were selected for N_2_O and NH_3_, a linear model for yield (as no plateau was observed even at the highest N rate), and a quadratic model for NUE. To address multicollinearity in the quadratic term, we centered the N rate variable. All models were validated for normality, homoscedasticity, independence of residuals, and absence of multicollinearity. The equations and detailed results have been added to the manuscript.

Comment 3:

The Discussion proposes three mechanisms for reduced N_2_O (slow release, physical barrier, prolonged NH_4_^+^ dominance). These remain speculative without inorganic-N data. Add at least three sampling dates for NH_4_^+^-N and NO_3_–-N (0–10 cm) or remove the unsupported claims.

Response:

We acknowledge this limitation. Unfortunately, we did not collect inorganic-N data during the experiment. Therefore, we have removed the speculative mechanisms regarding N_2_O reduction and instead cited relevant literature that supports the hypothesis that PCU reduces N_2_O emissions through slow nutrient release via a semi-permeable membrane.

Comment 4:

Include 2–3 recent open-field vegetable PCU studies to highlight novelty in the Discussion.

Response:

Thank you for this suggestion. We conducted a literature search using keywords such as "Chinese cabbage," "nitrogen," "ammonia volatilization," "greenhouse gas," "controlled-release nitrogen fertilizer," "PCU," and "NUE," limiting the timeframe to the past three years. We found no studies examining reduced PCU application rates on yield, N_2_O, and NH_3_ in Chinese cabbage. Extending the search to five years, we identified several related studies on cabbage and other vegetables using conventional urea reduction or organic substitutions. Our study is the first to combine reduced N rates with PCU technology in subtropical hilly dryland regions for Chinese cabbage, which we now emphasize in the Discussion.

Comment 5:

The price of PCU is higher than that of ordinary urea, and it is necessary to give input-output comparisons (fertilizer costs, yield changes, and environmental externalities) to enhance policy operability. A short paragraph is sufficient.

Response:

We sincerely thank the reviewer for this insightful comment. In direct response, our study provides a detailed input-output comparison that integrates fertilizer costs, yield impact, and environmental externalities. Specifically, while the direct fertilizer cost of the reduced PCU treatment (0.8PCU) was 44% higher than common urea (CU), the difference in pakchoi yield was not statistically significant. Most importantly, 0.8PCU significantly reduced NH₃ and N₂O emissions by 30.97% and 47.42%, respectively. By quantifying these environmental benefits into economic terms using established unit costs, we demonstrate that in a high environmental cost scenario (reflecting a more realistic societal burden), the net economic benefit of 0.8PCU narrows the gap with CU to within approximately 9%. This manageable economic trade-off, coupled with a dramatic reduction in nitrogen pollution, demonstrates that a 20% reduced application of PCU is a viable and sustainable strategy. We explicitly recommend policy instruments such as targeted subsidies for eco-friendly fertilizers to bridge the remaining cost gap and encourage farmer adoption.

Comment 6:

The time in the title of Fig 1 is "2022", which is inconsistent with the experimental year 2024 in the article.

Response:

We apologize for this oversight. The year in Figure 1 has been corrected to "2024".

Comment 7:

Check the English grammar throughout (such as Line 191: "it had no effect cabbage yield" missing preposition "on").

Response:

We have thoroughly reviewed the manuscript and corrected grammatical errors, including the missing preposition in Line 191. The language has been polished to improve clarity and accuracy.

We believe that these revisions have significantly improved the manuscript and thank you again for your insightful comments. We hope the revised version meets your approval.

Sincerely,

Yong Xie, Ph.D.

College of Agriculture, Guizhou University, Guiyang, China

E-mail: yongxie@gzu.edu.cn
