# Supplementary material for: Polymer-coated urea effects on yield and gaseous loss of nitrogen for planting Chinese cabbage
Source: PLoS One. 2025 Nov 26;20(11):e0337171. doi: 10.1371/journal.pone.0337171 (PMC12654927; doi:10.1371/journal.pone.0337171)
Supplement: Attachment — Submitted filename: Response to Reviewer.docx [file pone.0337171.s004.docx]

**Response to Reviewer**

Dear Reviewer,

We sincerely appreciate the time and effort you have dedicated to reviewing our manuscript. Your insightful comments have been invaluable in improving the quality of our paper. Below, we provide a point-by-point response to the questions you raised.

Response to Comment 1:

We are grateful for your insightful comment regarding the valuation of environmental costs. In response to your suggestion, we have revised the manuscript to provide appropriate references for the unit costs and calculation methods used in the cost-benefit analysis. Specifically, the social costs of N_2_O-N and NH_3_-N were based on Sutton et al. (2011), and the computational methodology followed Xie et al. (2020). The formula has been explicitly included in the Methods section, and the detailed results of the environmental cost calculations are now presented in the newly added Table 6. These modifications ensure full transparency and reproducibility of the assessment.

Response to Comment 2:

We thank you for highlighting this point. The sentence speculating that PCU “limits substrate supply to reduce N_2_O” has been removed from the discussion. Instead, we now state with supporting literature that the reduction in N_2_O emissions under PCU treatment may be attributed to enhanced nitrogen uptake by the crop and more favorable soil conditions, which effectively reduce the amount of nitrogen available for loss pathways. This revision is supported by a reference to relevant research (Yang et al. 2023). We believe this adjustment better aligns with the direct evidence and improves the scientific rigor of the discussion.

Thank you once again for your thorough review and constructive suggestions, which have greatly enhanced the clarity and accuracy of our manuscript. We look forward to your approval of the revised version.
