# Supplementary material for: Polymer-coated urea effects on yield and gaseous loss of nitrogen for planting Chinese cabbage
Source: PLoS One. 2025 Nov 26;20(11):e0337171. doi: 10.1371/journal.pone.0337171 (PMC12654927; doi:10.1371/journal.pone.0337171)
Supplement: Attachment — Submitted filename: Response to Reviewer(3).docx [file pone.0337171.s005.docx]

**Response to Reviewer**

Dear Reviewer,

Please find attached our revised manuscript, “[Polymer-coated urea effects on yield and gaseous loss of nitrogen for planting Chinese cabbage]” (ID: PONE-D-25-22820), submitted for your consideration.

We have thoroughly addressed the need for language polishing. Specifically, we have:

**1. We have corrected the specific language errors highlighted by the reviewers (e.g., in Lines 202 and 477).**

**2. We have performed a thorough proofreading of the entire manuscript to eliminate redundant phrasing and ensure concise and precise academic expression. Specific revisions and polish were made to the Abstract, Methods, and Conclusion sections.**

We are confident that these revisions have greatly improved the readability and quality of our work. The modifications are clearly highlighted in the manuscript for your easy reference.

We appreciate the chance to improve our paper and hope it is now suitable for publication.

Best regards,

Yong Xie, Ph.D.

College of Agriculture, Guizhou University, Guiyang, China

E-mail: yongxie@gzu.edu.cn
